# Effects of Intramolecular Distance between Amyloidogenic Domains on Amyloid Aggregation

**DOI:** 10.3390/ijms131012169

**Published:** 2012-09-25

**Authors:** Ahra Ko, Jin Ryoun Kim

**Affiliations:** Othmer-Jacobs Department of Chemical and Biomolecular Engineering, Polytechnic Institute of New York University, 6 MetroTech Center, Brooklyn, New York 11201, USA; E-Mail: koar324@hotmail.com

**Keywords:** amyloid, fibril, peptide aggregation, KFFE

## Abstract

Peptide/protein aggregation is implicated in many amyloid diseases. Some amyloidogenic peptides/proteins, such as those implicated in Alzheimer’s and Parkinson’s diseases, contain multiple amyloidogenic domains connected by “linker” sequences displaying high propensities to form turn structures. Recent studies have demonstrated the importance of physicochemical properties of each amino acid contained in the polypeptide sequences in amyloid aggregation. However, effects on aggregation related to the intramolecular distance between amyloidogenic domains, which may be determined by a linker length, have yet to be examined. In the study presented here, we created peptides containing two copies of KFFE, a simple four-residue amyloidogenic domain, connected by GS-rich linker sequences with different lengths yet similar physicochemical properties. Our experimental results indicate that aggregation occurred most rapidly when KFFE domains were connected by a linker of an intermediate length. Our experimental findings were consistent with estimated entropic contribution of a linker length toward formation of (partially) structured intermediates on the aggregation pathway. Moreover, inclusion of a relatively short linker was found to inhibit formation of aggregates with mature fibril morphology. When the results are assimilated, our study demonstrates that intramolecular distance between amyloidogenic domains is an important yet overlooked factor affecting amyloid aggregation.

## 1. Introduction

Peptide/protein aggregation is a molecular self-assembly process implicated in many amyloid diseases such as Alzheimer’s and Parkinson’s diseases (AD and PD, respectively) [1]. This molecular self-assembly process is affected by various molecular interactions, which determine aggregation kinetics and morphologies [2,3]. Amyloid aggregation involves two major steps, early stage aggregation into soluble oligomers and their subsequent conversion to amyloid fibrils [1]. Significant efforts have been put into identifying the physicochemical factors important in amyloid aggregation in order to better understand the molecular basis of amyloid diseases [4–8]. These factors include hydrophobicity, secondary structure propensity and charge state of amino acids contained in sequences [4–8]. Amyloid aggregation is a generic property of peptides and proteins [9], and amyloid aggregation of proteins and peptides of different primary sequences share similar molecular mechanisms and aggregate morphologies [10]. As such, molecular factors important in amyloid aggregation of a peptide/protein may directly be relevant to aggregation of other peptides/proteins associated with amyloid diseases. Biochemical and biophysical studies have further revealed various amino acid sequences displaying high propensities for amyloid aggregation [6,11,12]. Despite such recent progress, our understanding on the molecular aspects of amyloid aggregation is still significantly limited due to the complex nature of this self-assembly process, which involves various modes and types of molecular interactions [13–15].

A peptide/protein associated with amyloid diseases may contain multiple amyloidogenic domains [6,11]. For example, β-amyloid (Aβ) implicated in AD is composed of two hydrophobic amyloidogenic domains connected by a hydrophilic stretch of amino acids displaying a high propensity to form turn structures [7,16]. Similar placement of a turn-forming sequence between hydrophobic amyloidogenic domains is also found in α-synuclein (αS) implicated in PD [8]. The influences of mutations in amino acid sequences on amyloid aggregation have been extensively studied [4–8]. However, effects of intramolecular distance between amyloidogenic domains, which may be determined by the length of a linker region (*i.e.*, a region connecting amyloidogenic domains), on amyloid aggregation have yet to be studied.

In the study presented here, we aimed to examine effects of a linker length on aggregation of peptides containing multiple amyloidogenic domains. We were particularly interested in whether variation of a linker length can affect early stage aggregation as well as late stage fibrillization. To better determine effects of a linker length without being complicated by other sequence-associated factors, we chose a simple, four-residue amyloidogenic peptide domain, KFFE. KFFE was previously found to form β-sheet structured amyloid fibrils which were similar to those associated with many amyloid diseases [17]. Aggregation of KFFE was driven by intermolecular forces including electrostatic interactions between oppositely charged residues Lys and Glu, and hydrophobic interactions between high β-sheet-prone Phe-Phe [17]. π–π interactions between Phe residues in adjacent molecules may also promote aggregation of KFFE [17,18]. Results from previous studies suggest that the aforementioned physicochemical factors may play an important role in aggregation of peptides containing KFFE [17,19]. In our current study, we inserted several GS-rich sequences, which were carefully designed to display dissimilarity in length yet similarity in other physicochemical properties, between two identical KFFE domains. We then examined effects of the linker length on aggregation properties of the resulting peptides. Our study shows that there was an optimal linker length for rapid initiation of early stage aggregation of peptides containing two KFFE domains. Our experimental findings were consistent with estimated effects of a linker length on energetics associated with formation of (partially) structured intermediates on the aggregation pathway. We also provide evidence that formation of mature fibrils was inhibited by inclusion of a relatively short linker. Taken together, our results demonstrate an important yet overlooked role of intramolecular distance between amyloidogenic domains in amyloid aggregation and thus significantly contribute to a better understanding of the molecular basis of amyloid diseases.

## 2. Results and Discussion

In the present work, we characterized aggregation of peptides containing two KFFE domains connected by GS-rich linker sequences with different lengths yet similar physicochemical properties.

### 2.1. Design of Linker Sequences Connecting Two KFFE Domains

**Figure 1:**
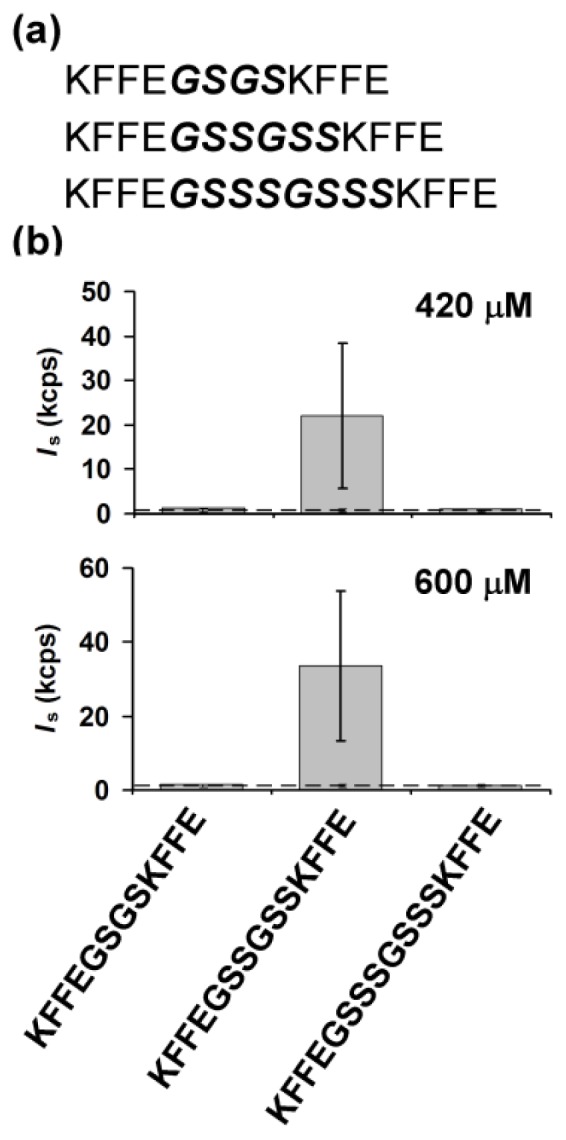
(**a**) The amino acid sequences of peptides tested in this study; (**b**) The scattered light intensities (*I*_s_) of samples containing peptides at 420 μM (top) and 600 μM (bottom) measured immediately after sample preparation. In (**a**), linker sequences are shown in italic bold. In (**b**), the dotted lines represent the scattered light intensities of buffer. The errors bars represent one standard deviation of at least two measurements.

The primary aim of this study was to examine effects of the intramolecular distance between KFFE domains on intermolecular aggregation of peptides containing these domains. To this end, we inserted several linker sequences in different lengths between KFFE domains. For these linker sequences, we searched for those which could vary the intramolecular distance between KFFE domains while minimizing variation of other physicochemical properties important in aggregation. As linker sequences, we chose GS-rich sequences which are widely used for flexible connection of multiple protein domains [20–22]. Gly and Ser are relatively neutral in terms of the aforementioned physicochemical properties important in amyloid aggregation. For example, Gly and Ser are uncharged at neutral pH, neither too hydrophobic nor too hydrophilic and low prone to form α-helices or β-sheets [23,24], and, as such, are highly suitable for linker residues in our study. Other non-flexible linker residues were not considered in our study since introduction of these residues may complicate interpretation of results by imposing other spatial constraints, such as those related to conformation [20,25]. Among many possible GS-rich sequences, we selected three linker sequences to create KFFEGSGSKFFE, KFFEGSSGSSKFFE and KFFEGSSSGSSSKFFE (Figure 1a) for the following reason; aggregation propensities of these peptides were predicted to be similar by aggregation predictors previously developed based on physicochemical properties of amino acids contained in a sequence [4,6] (e.g., aggregation scores calculated by Zyggregator = 0.558, 0.570 and 0.586 for KFFEGSGSKFFE, KFFEGSSGSSKFFE and KFFEGSSSGSSSKFFE, respectively). As such, we were able to examine effects of a linker length on aggregation of peptides containing KFFE domains without being complicated by other aggregation-determining factors. Molecular interactions may occur intramolecularly, for example, through electrostatic interactions between the oppositely charged *N*- and *C*-termini within a peptide sequence. However, intramolecular interactions present in the three different peptides, KFFEGSGSKFFE, KFFEGSSGSSKFFE and KFFEGSSSGSSSKFFE are unlikely to exert any significant differential effects on intermolecular aggregation of these peptides for the following reasons: i) end-to-end distances of the three peptides were estimated to be similar (*i.e*., ~17, ~18 and ~19 Å, respectively, with increasing linker length) when they were calculated with a worm-like chain model as described previously [26]; ii) the previous computational study suggested that intramolecular interaction energies of KFFE were similar for two different conformations formed at 300 K and 700 K [27].

### 2.2. Effects of a Linker Length on Early Stage Aggregation of Peptides

For sensitive monitoring of early stage aggregation of peptides in solution state, laser light scattering was employed. The scattered light intensities, which are proportional to the apparent weight-averaged molecular weight of particles in solution [28], were used as a primary measure of extent of aggregation. Interestingly, when freshly prepared at 420 μM, only samples containing KFFEGSSGSSKFFE displayed significant scattered light intensities (*i.e.*, ~16-fold higher than those of buffer, Figure 1b). Scattered light intensities of samples containing KFFEGSGSKFFE or KFFEGSSSGSSSKFFE were not significantly different from those of buffer (Figure 1b). A similar trend was also observed when early stage aggregation was monitored immediately after preparation of fresh peptide samples at 600 μM (Figure 1b). Note that when freshly prepared, all peptide samples were optically clear and lacked any significant insoluble aggregates at least for ~5–6 h. Taken together, our findings indicate that early stage soluble aggregation occurred most rapidly with KFFEGSSGSSKFFE. In other words, there was an optimal linker length for rapid initiation of aggregation of peptides containing two KFFE domains. The scattered light intensities of samples were not sufficiently high for reliable evaluation of Z-average hydrodynamic diameters of peptide aggregates, and therefore such evaluation was not performed. All three peptides including KFFEGSSGSSKFFE exhibited mostly disordered structures in solution as determined by circular dichroism (CD) spectroscopy (Figure S1), suggesting that early stage soluble aggregation of KFFEGSSGSSKFFE was not accompanied by significant formation of regular secondary structures (*i.e.*, α-helix and β-sheet). Broad maxima at ~215–220 nm detected in the CD spectra are indicative of the presence of local, residual poly(pro)II helical structures in these peptide samples (Figure S1), similar to other proteins in disordered states [29–31]. The magnitude of the maximum was found to decrease with increasing linker length (Figure S1), presumably due to a resultant increase in the number of flexible residues (*i.e.*, Ser) within a peptide sequence [32]. The scattered light intensities of all three samples significantly increased after 4 days of incubation at 37 °C with constant stirring (*i.e.*, ~14, ~350 and ~13 kcps for KFFEGSGSKFFE, KFFEGSSGSSKFFE and KFFEGSSSGSSSKFFE, respectively, at 420 μM each), indicating that all these peptides aggregated under our experimental condition.

### 2.3. Effects of a Linker Length on the Morphology of Peptide Aggregates

**Figure 2:**
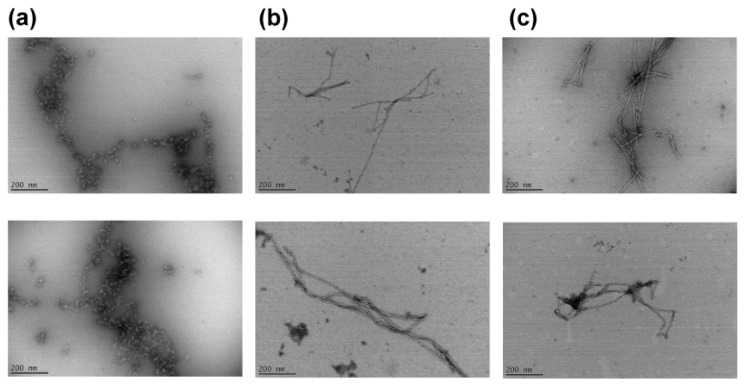
Representative transmission electron microscopy (TEM) images of samples containing (**a**) KFFEGSGSKFFE, (**b**) KFFEGSSGSSKFFE and (**c**) KFFEGSSSGSSSKFFE after 4 days of incubation at 37 °C with constant stirring at 250 rpm using a magnetic stir bar. Peptide concentrations in samples during incubation were 420 μM. Scale bars represent 200 nm.

We then examined morphologies of peptide aggregates formed after 4 days of incubation using transmission electron microscopy (TEM). Aggregates formed by KFFEGSSGSSKFFE and KFFEGSSSGSSSKFFE were found to display mature fibrillar morphology (Figure 2b,c). Interestingly, curvy, rather than mature fibrillar, aggregates were detected in samples containing KFFEGSGSKFFE (Figure 2a). Taken together, our findings indicate that inclusion of a relatively short linker may inhibit formation of mature fibrils. We also examined aggregation of the peptide samples using fluorescence of thioflavin T (ThT), a fluorescent dye specific for amyloid β-sheet structures [33]. ThT fluorescence of the three samples was not significantly different from that of buffer during 4 days of incubation at 37 °C with constant stirring. These findings indicate that i) aggregates formed by the three peptides were mostly ThT-negative and/or ii) only small amounts of ThT-positive fibrils were formed in these samples. Secondary structures of all peptide samples remained mostly disordered after 4 days of incubation as determined by CD (Figure S1), suggesting that molecular entities lacking regular secondary structures (*i.e.*, α-helix and β-sheet) represented the dominant fractions of samples during incubation.

### 2.4. Entropic Contribution of a Linker Length toward Formation of (Partially) Structured Intermediates

**Figure 3:**
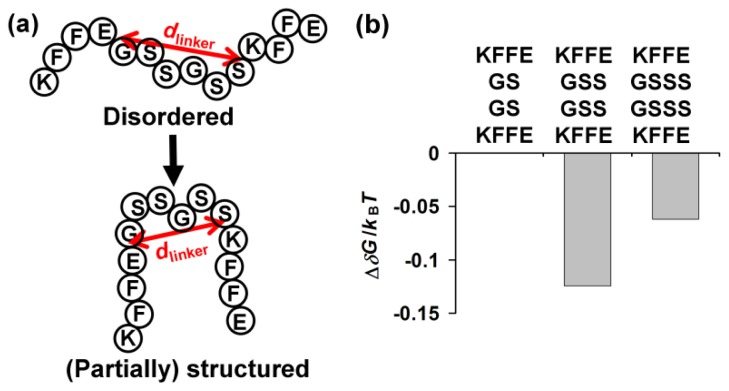
(**a**) A schematic of the proposed formation of (partially) structured intermediates by a peptide from its disordered states; (**b**) ΔδG, the change in δG (*i.e.*, the free energy change from the disordered to (partially) structured states) caused by increasing a linker length when *d*_linker_ in the (partially) structured state (*i.e.*, *d*_linker, pss_) = 13.5 Å. In (**a**), the symbol *d*_linker_ represents the mean distance between the ends of a linker. Formation of (partially) structured intermediates is exemplified with KFFEGSSGSSKFFE. In (**b**), the value of δG for KFFEGSGSKFFE was used as a reference and therefore ΔδG for KFFEGSGSKFFE = 0. *k*_B_: the Boltzmann constant.

Previous biochemical and biophysical studies revealed several physicochemical factors determining aggregation [4–8]. However, our results demonstrate that an additional factor may as well determine amyloid aggregation behaviors. This additional factor may be related to energetics associated with an initial structural rearrangement leading to high order self-assembly as follows: we postulated that aggregation of KFFEGSGSKFFE, KFFEGSSGSSKFFE and KFFEGSSSGSSSKFFE might occur through formation of structured, at least partially, intermediates (Figure 3a) as was the case with aggregation of many other amyloidogenic peptides [34–36]. These (partially) structured intermediates represent molecular entities compatible with high order assembly during aggregation [34–36]. Note that the dominant fractions of samples containing KFFEGSGSKFFE, KFFEGSSGSSKFFE or KFFEGSSSGSSSKFFE were structurally disordered as described above, suggesting that these putative intermediates should represent only minor populations. We then analyzed entropic effects of the intramolecular distance between KFFE domains on formation of (partially) structured intermediates from disordered states. Specifically, we sought to evaluate the change in the free energy of formation of (partially) structural intermediates caused by variation in the linker length using polymer theories [37]. Equations were previously derived for determination of changes in folding energy of a protein as a function of the length of its constituting loop [37]. A similar approach was applied to assess effects of a linker length on formation of (partially) structured intermediates with assumption that linkers (e.g., GSGS) connecting amyloidogenic domains (*i.e.*, KFFE) behave as worm-like chains (see supplementary material for details). Note that a worm-like chain model was found to successfully describe conformational behaviors of loops connecting structural domains of a protein [37,38], justifying the use of this model for our study.

Interestingly, the free energy change from the disordered to (partially) structured states (abbreviated by δG) was found to be the lowest with KFFEGSSGSSKFFE when the mean distance between the ends of a linker (abbreviated by *d*_linker_ in Figure 3a) was assumed to be 13.5 Å in the (partially) structured state (Figure 3b). This analysis is consistent with our finding that aggregation occurred most rapidly with KFFEGSSGSSKFFE (Figure 1b), provided that significant formation of (partially) structured intermediates is a prerequisite for the onset of detectable peptide self-assembly. Formation of (partially) structured intermediates was found to be energetically less favorable when a longer linker (*i.e.*, GSSSGSSS) than GSSGSS was included between KFFE domains (Figure 3b). This is because the occurrence of structural reorganization (e.g., close contacts between the connected KFFE domains) leading to the formation of (partially) structured intermediates is entropically less favored with a longer linker. It should also be noted that structural flexibility of a linker represented by its persistence length may also directly affect energetics associated with formation of (partially) structured intermediates (see Equations 1 and 2 in supplementary material for details). While the entropic disadvantage of inclusion of a relatively long linker may delay the onset of detectable aggregation of KFFEGSSSGSSSKFFE (Figure 1b), formation of mature fibrils by this peptide was still permitted (Figure 2c). Note that the value of 13.5 Å set for *d*_linker_ in the (partially) structured state (*i.e.*, *d*_linker, pss_) is close to the distance between β sheets in amyloid fibrils (*i.e.*, ~10 Å) [39,40] given consideration of potential structural heterogeneity of (partially) structured intermediates. The implication is that these intermediates may have a β sheet-like conformation to some extent. The end-to-end distance of the GSGS linker may not be long enough to span the optimal distance between KFFE domains for aggregation to form fibrils. This structural restraint might inhibit formation of mature fibrils by KFFEGSGSKFFE while allowing it to self-assemble into curvy aggregates (Figure 2a). Similar to isolated KFFE domains during their self-assembly, the three peptides we tested may assemble in an anti-parallel orientation, which may primarily be driven by electrostatic interactions between oppositely charged Lys and Glu residues from adjacent molecules [17,27]. However, the possibility of these three peptides to assemble in other orientations may not be completely excluded as discussed previously [19].

## 3. Experimental Section

### 3.1. Materials

Peptides (*i.e.*, KFFEGSGSKFFE, KFFEGSSGSSKFFE and KFFEGSSSGSSSKFFE) were synthesized using solid-phase chemistry, purified using reverse-phase HPLC by Genscript (Piscataway, NJ, USA). All peptides were lyophilized after purification. The identities of peptides were confirmed by MALDI-TOF mass spectrometry. All other chemicals were purchased from Fisher Scientific (Pittsburg, PA, USA) unless otherwise stated.

### 3.2. Sample Preparation

For preparation of samples, lyophilized peptides were dissolved in phosphate-buffered saline with azide (PBSA, 10 mM Na_2_HPO_4_/NaH_2_PO_4_, 150 mM NaCl, 0.02% (*w*/*v*) NaN_3_, pH 7.4). The peptide solutions were subsequently filtered with 0.45 μm syringe filters to remove any remaining large aggregates, and the concentrations of the filtered peptide solutions were measured using a bicinchoninic acid protein assay according to the manufacturer’s protocol (Pierce Biotechnology, Rockford, IL, USA). The individual peptide concentrations were then immediately adjusted to 420 μM by addition of 1X PBSA unless otherwise mentioned, and the samples were subsequently incubated at 37 °C with constant stirring at 250 rpm using a magnetic stir bar to initiate aggregation.

### 3.3. Laser Light Scattering

Aggregation of peptides in solution was monitored by laser light scattering using the Zetasizer Nano-S system (Malvern Instruments Ltd., Malvern, UK). Peptide samples were placed in quartz cuvettes and intensities of scattered light at 633 nm were then measured at 90° relative to the incident light at the same wavelength.

### 3.4. Transmission Electron Microscopy (TEM)

The aliquot (5 μL) of a sample was placed on carbon membrane coated, glow discharged grids and negatively stained with 3% uranyl acetate in deionized water for 5 min. The samples were imaged on a Philips CM12 Transmission Electron Microscope (FEI Corp.: Hillsboro, OR, USA) at 120 kV with a 4 k × 2.67 k GATAN digital camera located at the Image Core Facility of the Skirball Institute of Biomedical Sciences, NYU School of Medicine.

### 3.5. Circular Dichroism (CD) Spectroscopy

Secondary structures of peptides in solutions were determined using CD, collected using a Jasco J-815 spectropolarimeter in the far-UV range with a 0.1 cm pathlength cuvette. Ellipticity of samples at each wavelength was measured immediately after 10-fold dilution by PBSA. The spectrum of the background (buffer only) was also measured and then subtracted from the sample spectrum.

### 3.6. Thioflavin T (ThT) Fluorescence

Twenty μL of peptide sample was mixed with 10 μL of 0.1 mM ThT solution in water and 170 μL of PBSA per 200 μL of the final volume. ThT fluorescence of samples was then immediately measured using a Photon Technology QuantaMaster QM-4 spectrofluorometer. Excitation wavelength was 440 nm and emission was monitored at 485 nm.

## 4. Conclusions

Results from our study suggest that (1) the intramolecular distance between KFFE domains may affect the onset of early stage aggregation as well as morphology of aggregates; (2) there was the optimal intramolecular distance between KFFE domains, which was corresponding to the end-to-end distance of the GSSGSS linker (*i.e.*, ~13.5 Å in the (partially) structured state), for rapid initiation of aggregation; and (3) our experimental findings were consistent with the estimated entropic contribution of a linker length toward formation of (partially) structured intermediates. Taken altogether, our study demonstrates important yet overlooked effects of the length of a linker connecting multiple amyloidogenic domains on amyloid aggregation. Results from our study also provide insight into the role of a similar linker in aggregation of naturally existing peptides and proteins implicated in amyloid diseases. For example, differences in lengths of linker regions of various proteins/peptides containing multiple amyloidogenic domains [4,6–8,41] may further differentiate aggregation propensities, which are also affected by other factors such as physicochemical properties of amino acid sequences [4,6–8,41]. It should also be noted that a compound capable of binding to a linker region has a high potential to modulate amyloid aggregation by affecting linker’s structural flexibility, which may determine energetics associated with formation of (partially) structured intermediates as described above. Such compounds may be considered as an important class of aggregation modulators for amyloid diseases. Similar aggregation modulation may also be mediated by interactions between a linker region and lipid headgroups, and such interactions are involved in important molecular events associated with the Aβ linker region and lipid membranes [42,43].

## Supplementary Information

### Theoretical analysis on the free energy of formation of (partially) structured intermediates as a function of linker length

For our analysis, linkers (e.g., GSGS) connecting amyloidogenic domains (*i.e.*, KFFE) were considered to behave as worm-like chains. The normalized distribution function of an end-to-end vector of a worm-like loop depends on the contour length (*l*_c_) and the persistence length (*l*_p_) of a loop [44]. The same form of the normalized distribution function was assumed to be applicable for the end-to-end vector of a linker connecting two KFFE domains. For short unstructured peptides, *l*_c_ = 3.8 Å × *l* where *l* is the number of peptide bonds present in an amino acid sequence [44] and *l*_p_ can be approximated by 3.04 Å [37]. The end-to-end vector of a linker is also restrained to a distribution function depending on the conformational state (*i.e.*, (partially) structured vs. unstructured states) of a peptide containing two KFFE domains. For example, the end-to-end vector of a linker (*d*_linker_) should display relatively small, restricted fluctuations around the mean displacement, *d*_linker, pss_, when a peptide exists in the (partially) structured state. In contrast, no such restriction on the end-to-end vector of a linker may be found when a peptide is in the unstructured state. δG, the free energy of formation of (partially) structured intermediates of a peptide containing KFFE domains connected by a linker can then be given using fractions of allowed conformations in the (partially) structured and unstructured states, and the aforementioned distribution functions of the end-to-end vector of a linker [37]. The change in δG (*i.e.*, ΔδG) caused by variation in a linker length can subsequently be expressed as the following:

(1) $$\begin{array}{c} \Delta \delta G/k_{\text{B}}T=(3/2)\;\text{ln }l+3{d_{\text{linker},\text{pss}}}^{2}/(4\times 3.04\times 3.8l)-\text{ln}[1-f(d_{\text{linker},\text{pss},}l)]-(3/2)\;\text{ln }l_{0}-\\ 3{d_{\text{linker},\text{pss}}}^{2}/(4\times 3.04\times 3.8l_{0})+\text{ln}[1-f(d_{\text{linker},\text{pss}},l_{0})] \end{array}$$

(2) $$\begin{array}{c} f(d_{\text{linker},\text{pss}},l)=5\times 3.04/(4\times 3.8l)-2\times {\left(d_{\text{linker},\text{pss}}\right)}^{2}/{\left(3.8l\right)}^{2}+\\ 33\times {\left(d_{\text{linker},\text{pss}}\right)}^{4}/(80\times 3.04\times {\left(3.8l\right)}^{3})+79\times 3.04^{2}/(160\times {\left(3.8l\right)}^{2})+329\times 3.04\times \\ {\left(d_{\text{linker},\text{pss}}\right)}^{2}/(120\times {\left(3.8l\right)}^{3})-6799\times {\left(d_{\text{linker},\text{pss}}\right)}^{4}/(1600\times {\left(3.8l\right)}^{4})+3441\times \\ {\left(d_{\text{linker},\text{pss}}\right)}^{6}/(2800\times 3.04\times {\left(3.8l\right)}^{5})-1089\times {\left(d_{\text{linker},\text{pss}}\right)}^{8}/(12800\times 3.04^{2}\times {\left(3.8l\right)}^{6}) \end{array}$$

where *k*_B_ = the Boltzmann constant, *T* = temperature in Kelvin, *l* = the number of peptide bonds present in an amino acid sequence, *l*_0_ = *l* at the reference state, *d*_linker, pss_ = the mean displacement in the unit of Å between the ends of a linker when a peptide exists in the (partially) structured state.

The above Equations 1 and 2 are in a similar form as those previously derived for the change in the folding free energy of a protein caused by variation in a length of a loop connecting structural domains of a protein [37].

Figure S1 Circular dichroism (CD) spectra of samples containing KFFEGSGSKFFE (black squares), KFFEGSSGSSKFFE (red circles) and KFFEGSSSGSSSKFFE (blue triangles) at day 0 (empty symbols) and day 4 (filled symbols). Peptide samples at 420 μM each were incubated at 37 °C with constant stirring at 250 rpm using a magnetic stir bar.
