# Supplementary figures and images for: Effects of Intramolecular Distance between Amyloidogenic Domains on Amyloid Aggregation
Source: Int J Mol Sci. 2012 Sep 25;13(10):12169–81. doi: 10.3390/ijms131012169 (PMC3497264; doi:10.3390/ijms131012169)

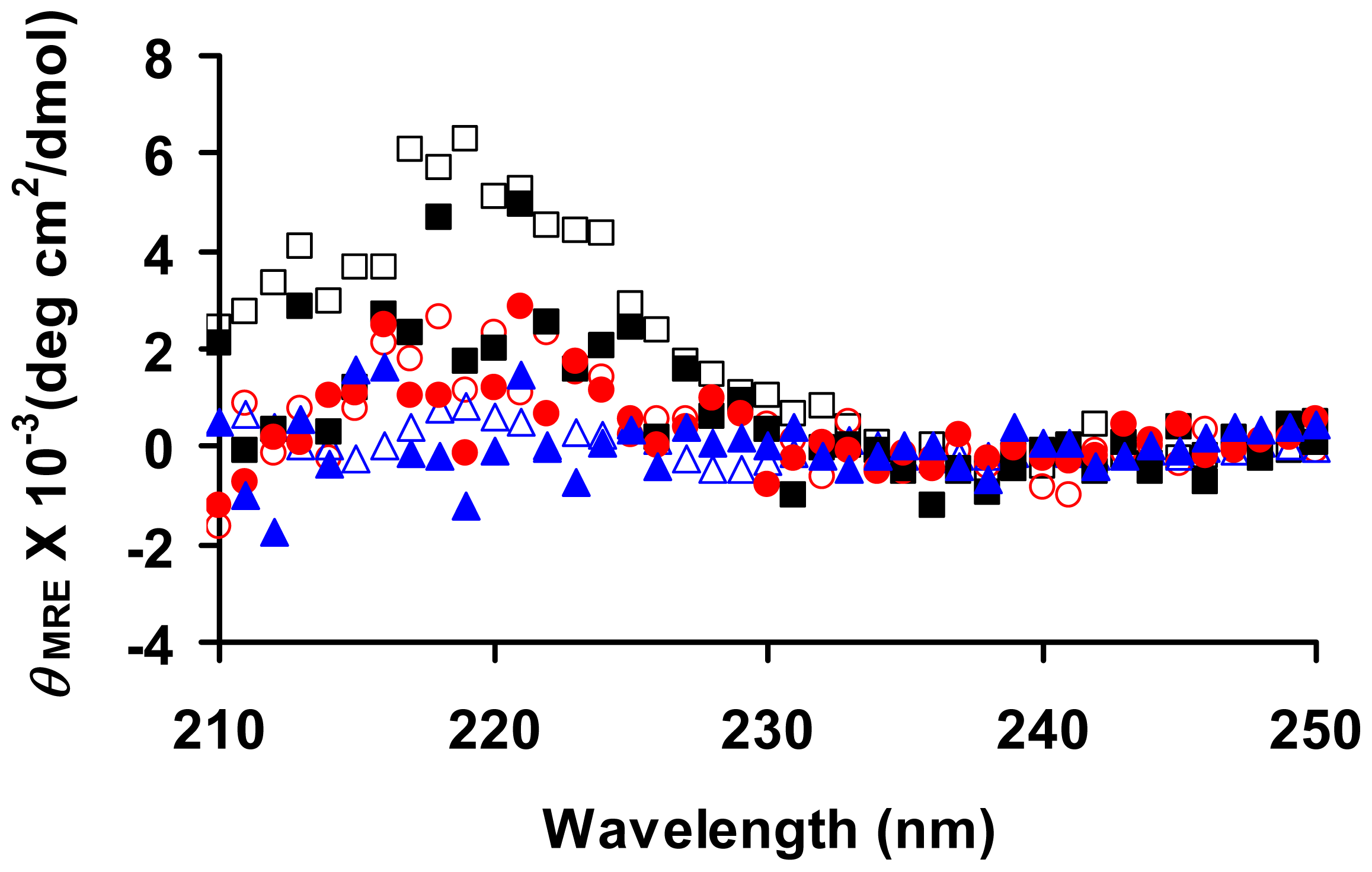

Supplement: Figure S1 — Circular dichroism (CD) spectra of samples containing KFFEGSGSKFFE (black squares), KFFEGSSGSSKFFE (red circles) and KFFEGSSSGSSSKFFE (blue triangles) at day 0 (empty symbols) and day 4 (filled symbols). Peptide samples at 420 μM each were incubated at 37 °C with constant stirring at 250 rpm using a magnetic stir bar. [file ijms-13-12169s1.tif]
